# Tick bites in different professions and regions: pooled cross-sectional study in the focus area Bavaria, Germany

**DOI:** 10.1186/s12889-021-12456-3

**Published:** 2022-02-04

**Authors:** Louisa Schielein, Linda Tizek, Tilo Biedermann, Alexander Zink

**Affiliations:** 1 Department of Dermatology and Allergy, Technical University of Munich, School of Medicine, Biedersteiner Strasse 29, 80802 Munich, Germany; 2 Division of Dermatology and Venereology, Department of Medicine Solna, Karolinska Institutet, Stockholm, Sweden

**Keywords:** Tick bite, Prevalence, Profession, Region, Bavaria, Tick-associated risk

## Abstract

**Background:**

As the vector-borne diseases tick-borne encephalitis (TBE) and Lyme borreliosis (LB) are common in Germany and transmitted by tick bites, the aim of this study was to assess differences in the number of tick bites in various professions and regions across southern Germany to evaluate the differences in tick-associated risk.

**Materials and methods:**

The analysis is based on three cross-sectional studies that were conducted in 2016 and 2017 in two real-life settings and in one medical setting in Bavaria. All participants filled in a paper-based questionnaire about their history with tick bites. Only adult participants (≥ 18 years) were included in this study.

**Results:**

Overall, 3503 individuals (mean age 50.8 ± 15.2 years, median age 53.0 ± 12.2 years, 54.0% female) were included. Of these, 50% worked in an outdoor profession and 56% lived in environs. Around 70% of participants reported at least one previous tick bite. In comparison to indoor workers, forestry workers (OR = 2.50; 95% CI: 1.10–5.68) had the highest risk for a tick bite followed by farmers (OR = 1.22; 95% CI: 1.01–1.47). Furthermore, people living in rural areas (OR = 1.97, 95% CI:1.49–2.59) and environs (OR = 1.98, 95% CI: 1.54–2.55) were twice as likely to have a previous tick bite than people living in urban areas. In general, slightly more tick bites were reported by people living in eastern Bavaria.

**Conclusion:**

Rising numbers of TBE and LB indicate the need for further prevention strategies, which should focus on outdoor professions with a higher risk and people living in environs and rural areas.

## Background

In Europe, the most common vector-borne diseases are Lyme borreliosis (LB) and tick-borne encephalitis (TBE), both being transmitted by ticks of the species *Ixodes ricinus* [1–3]. In Germany, TBE and LB are also widespread, with the highest reported incidence of TBE being in the two southern federal states of Bavaria and Baden-Wuerttemberg. LB, however, is nearly equally distributed across Germany [2, 4, 5]. In 2020, a new peak of reported cases of TBE and LB was recorded, indicating a continuing need to improve prevention [4, 6]. Considering that ticks are typically concentrated in areas with suitable climate conditions and presence of hosts, the typical habitats of *I. ricinus* tend to be lowland, humid biotopes like unmanaged grasslands, heaths, forest edges, woodlands, and broad-leaf forests [7, 8]. In central Europe, ticks are usually active between March and November, with peak activity in the warm and humid months of May, June, and September. However, they can also be found during warmer winter days, as ticks can be active in temperatures close to the freezing point [9–11].

Investigations in the last few years showed that in Germany the prevalence of human-derived ticks infected with LB fluctuates between 17.1–26.0% depending on the area and that less than 1% of ticks carry the TBE-virus [12, 13]. Those TBE infested ticks are not evenly distributed across the landscape but tend to be found in small natural foci with a higher proportion of ticks carrying the TBE virus, whereas outside of those focus areas the number of TBE infected ticks can be close to zero [3, 14, 15]. After a bite, the associated risk of manifested Lyme disease is assumed to be between 0.3–1.4%, whereas the risk of manifested TBE cannot be estimated, as most infections go unnoticed [16–18]. Therefore, the prevalence of both diseases is difficult to estimate correctly and underlies small-scale fluctuations. Additionally, LB is not a notifiable disease in all of Germany. For better risk estimation, unconventional methodologies like Google search volume analyses can help monitor disease distribution or local outbreaks [19].

TBE and LB can cause severe long-term health issues. TBE results in neurological complications in up to 58% of cases and in death in 0.5–2.0% of cases. LB can progress into more severe forms like neuroborreliosis, Lyme-arthritis or Lyme-carditis [20–22]. Highly effective vaccines are available to prevent TBE and its complications and are recommended for inhabitants of risk areas and workers of occupations with a higher risk for infection, such as forestry workers, farmers, and exposed laboratory staff [18, 23, 24]. Since there is no effective vaccine for LB currently available, prevention strategies and increasing awareness are of high importance [25]. Prevention measures should be adapted to the specific needs and knowledge of high-risk groups and to local geographic circumstances for effective implementation. Furthermore, it is necessary to identify regions or people with a higher medical need for prevention. As a complement to typical medical settings, real-life settings can provide convenient access to healthcare and thereby include a high number of individuals of a target population. Thus, the study was conducted in two real-life settings and one typical medical setting to examine the history of tick bites in various professions and regions.

## Methods

### Data collection

This pooled-data analysis included data from three cross-sectional studies. The first and largest study was performed at the “Bavarian Central Agricultural Festival” (Bayerisches Zentral-Landwirtschaftsfest [ZLF]), which took place between 17 and 25 September 2016 [26, 27]. The ZLF takes place every four years as part of the Munich Oktoberfest and has around 300,000 visitors from across Germany, especially from Bavaria. In cooperation with the German “Social Insurance for Agricultural, Forestry and Horticulture (Sozialversicherung für Landwirtschaft, Forsten und Gartenbau), visitors were offered a free health examination on-site [26, 28]. The second study was performed in the first quarter of 2017 in rural areas of the Bavarian Forest (the areas of Cham, Freyung-Grafenau, Passau, and Regen) [29, 30]. Participants were recruited from 19 private practices that specialize in general medicine (*n* = 10), internal medicine (*n* = 2), orthopedics (*n* = 6), or surgery (*n* = 1). To reduce selection bias, both patients and their accompanying person were included in the study [30]. The third study was conducted at the annual winter meeting of three hunting associations in Bavaria (Wolfratshausen, Landsberg am Lech, and Freising) in December 2016 and at the international exhibition for hunting and fishing in January 2017, which is held annually in the greater Munich area [31].

In all three studies, participants were asked to fill in a paper-based questionnaire including questions on general data (gender, age, place of residence, and profession), average time spent outdoors in summer and winter, and the number of prior tick bites. According to the questionnaire, participants were classified as farmers, forestry workers, horticulturists, other outdoor workers (e.g. construction worker, council worker viniculturist, hunter, fully qualified groom and riding instructor, mailman), or indoor workers. If a participant indicated to have several professions, participants were classified according to the first stated profession.

To examine whether there were regional differences among participants living in Germany, the reported postal code was used to establish three area types. The area types were divided into: (1) urban areas (cities with at least 100,000 inhabitants); (2) environs (urban areas with at least 50% of the population living in medium-sized towns [20,000–99,999 inhabitants] or areas with at least 150 inhabitants/km^2^); and (3) rural areas (sparsely populated areas with less than 50% of the population living in medium-sized towns or less than 100 inhabitants/km^2^). These categories were selected according to the criteria from the German Federal Institute for Research on Building, Urban Affairs and Spatial Development and because they were previously used in one of the partial studies of this pooled study [27, 29]. Individuals had to be 18 years or older and provide written informed consent to participate in this study. Individuals who did not report hours spent outdoors or did not live in Germany were excluded from the pooled-data analysis. All three studies were approved by the ethics committee of the Medical Faculty of the Technical University of Munich (Reference: 385/16 s [27]; 584/16 s [30]; 405/15 s [31]).

### Statistical analyses

Descriptive data were generated for all variables. To assess differences in the study population, Kruskal-Wallis-Tests were applied with Mann-Whitney-U post hoc tests. For each area with at least 30 participants, the proportion of people who were affected by one or more tick bites were estimated and 95% Bootstrap confidence intervals (CI) were calculated (1000 samples). To assess factors that influence the likelihood of tick bites, univariate and multiple logistic regression models were applied. In this analysis, area type (urban, environs, or rural), age groups (18–34 years, 35–44 years, 45–54 years, 55–64 years, or ≥ 65 years), gender, profession (farmer, forestry worker, horticulturist, other outdoor worker, or indoor worker), and time spend outside during summer and winter (<1 h, 1-3 h, >3-6 h, >6 h) were selected as explanatory variables. Each variable that was significant in the univariate model was added to the multiple logistic regression model via the enter method. The adjusted odds ratios (ORs) were calculated with 95% CI. For selected factors, a multiple imputation method generated a total of five imputations to account for missing data. IBM SPSS 26 was used for data management and statistical analyses. Spatial analyses were performed using a geographic information system (QGIS 2.14.22; QGIS.ORG, Grüt, Switzerland) and geodata from the German Federal Agency for Cartography and Geodesy [32] that describe the administrative boundaries.

## Results

A total of 3503 participants were included in the analysis. The participant mean age was 50.8 ± 15.2 years (median 53.0 ± 12.2 years, range 18–90 years) and the proportion of women (54.0%) was slightly higher than that of men (45.7%). Participants lived in 160 different districts across Germany, with the vast majority (94.0%) located in the federal state of Bavaria. Based on the reported postal codes, most individuals lived in environs (56.0%) followed by rural areas (32.0%) and urban areas (9.8%). With 1741 (49.8%) participants having an outdoor profession (farmer, forestry worker, horticulturist, and other outdoor worker), the proportion of outdoor workers was higher than that of indoor workers (38.5%, *p* < 0.001, Table 1).

**Table 1:** Baseline characteristics of participants separated by the number of tick bites

	Total (***n*** = 3503)	Tick bite (***n*** = 2466)	< 5 bites (***n*** = 1319)	5–10 bites (***n*** = 435)	> 10 bites (***n*** = 347)	Missing (***n*** = 365)
**Gender**						
Female	1892 (54.0%)	1334 (70.5%)	739 (56.0%)	238 (54.7%)	161 (46.4%)	196 (53.7%)
Male	1601 (45.7%)	1125 (70.3%)	574 (43.5%)	196 (45.1%)	186 (53.6%)	169 (46.3%)
*Missing*	*10 (0.3%)*	*7 (70,0%)*	*6 (0.5%)*	*1 (0.2%)*	*0 (0.0%)*	*0 (0.0%)*
**Age**						
18–34 years	629 (18.0%)	443 (70.4%)	294 (22.3%)	81 (18.6%)	31 (8.9%)	37 (10.1%)
35–44 years	386 (11.0%)	258 (66.8%)	160 (12.1%)	48 (11.0%)	27 (7.8%)	23 (6.3%)
45–54 years	859 (24.5%)	615 (71.6.%)	335 (25.4%)	113 (26.0%)	95 (27.4%)	72 (19.7%)
55–64 years	944 (26.9%)	659 (69.8%)	300 (22.7%)	116 (26.7%)	122 (35.2%)	121 (33.2%)
≥ 65 years	635 (18.1%)	454 (71.5%)	213 (16.1%)	74 (17.0%)	71 (20.5%)	96 (26.3%)
*Missing*	*50 (1.4%)*	37 (74.0%)	*17 (1.3%)*	*3 (0.7%)*	*1 (0.3%)*	*16 (4.4%)*
**Profession**						
Farmer	1452 (41.5%)	1068 (73.6%)	502 (38.1%)	184 (42.3%)	177 (51.0%)	205 (56.2%)
Forest worker	49 (1.4%)	42 (85.7%)	15 (1.1%)	12 (2.8%)	10 (2.9%)	5 (1.4%)
Horticulturist	160 (4.6%)	96 (60.0%)	57 (4.3%)	15 (3.4%)	7 (2.0%)	17 (4.7%)
Other type of outdoor worker	80 (2.3%)	53 (66.3%)	24 (1.8%)	15 (3.4%)	10 (2.9%)	4 (1.1%)
Indoor worker	1350 (38.5%)	891 (66.0%)	521 (39.5%)	135 (31.0%)	104 (30.0%)	131 (35.9%)
*Not reported*	*412 (11.8%)*	316 (76.7%)	*200 (15.2%)*	*74 (17.0%)*	*39 (11.2%)*	*3 (0.8%)*
**Hours spent outside (summer)**						
< 1 h	240 (6.9%)	136 (56.7%)	76 (5.8%)	21 (4.8%)	16 (4.6%)	23 (6.3%)
1–3 h	862 (21.6%)	620 (71.9%)	371 (28.1%)	115 (26.4%)	66 (19.0%)	68 (18.6%)
> 3–6 h	1159 (33.1%)	825 (71.2%)	438 (33.2%)	143 (32.9%)	128 (36.9%)	116 (31.8%)
> 6 h	1242 (35.5%)	885 (71.3%)	434 (32.9%)	156 (35.9%)	137 (39.5%)	158 (43.3%)
**Hours spent outside (winter)**						
< 1 h	534 (15.2%)	363 (68.0%)	217 (16.5%)	66 (15.2%)	35 (10.1%)	45 (12.3%)
1–3 h	1522 (43.4%)	1068 (70.2%)	600 (45.5%)	190 (43.7%)	146 (42.1%)	132 (36.2%)
> 3–6 h	841 (24.0%)	602 (71.6%)	309 (23.4%)	106 (24.4%)	89 (25.6%)	98 (26.8%)
> 6 h	511 (14.6%)	364 (71.2%)	165 (12.5%)	64 (14.7%)	65 (18.7%)	70 (19,2%)
*Not reported*	*95 (2.7%)*	69 (72.6%)	*28 (2.1%)*	*9 (2.1%)*	*12 (3.5%)*	*20 (5.5%)*
**Area types**						
Urban area	342 (9.8%)	185 (54.1%)	120 (9.1%)	29 (6.7%)	13 (3.7%)	23 (6.3%)
Environs	1961 (56.0%)	1417 (72.3%)	722 (54.7%)	274 (63.0%)	216 (62.2%)	205 (56.2%)
Rural areas	1121 (32.0%)	812 (72.4%)	447 (33.9%)	127 (29.2%)	109 (31.4%)	129 (35.3%)

### Comparison regarding the probability of tick bites

Overall, 70.4% of participants reported at least one previous tick bite, with no significant difference regarding gender (female: 70.5% vs. male: 70.3%, *p* = 0.878) and age groups (*p* = 0.485). Forestry workers not only had the highest proportion of people with a previous tick bite (85.7%) but also the highest proportion of individuals with more than 10 previous tick bites (23.8%). With 73.6%, farmers were the second most affected profession followed by other outdoor workers (66.3%). The multivariate analysis revealed that people living in environs (OR = 1.98, 95% CI: 1.54-2.55) and people living in rural areas (OR = 1.97, 95% CI: 1.49; 2.59) were twice as likely to have a previous tick bite than people living in urban areas. Furthermore, farmers (OR = 1.22, 95% CI: 1.01; 1.46) and forestry workers had a significantly higher risk (OR = 2.50, 95% CI: 1.10; 5.68) than indoor workers, whereas horticulturists (OR = 0.89, 95% CI: 0.63-1.26) and other outdoor workers (OR = 0.92, 95% CI: 0.56; 1.51) showed a smaller risk in comparison to indoor workers. The probability of a tick bite was also influenced by the amount of time that participants spent outside during summer. In comparison to people who spend less than one hour outside, people spending one to three hours outside were twice as likely to have a tick bite (OR = 1.93, 95% CI: 1.43-2.60, Table 2).

**Table 2:** Results of the univariate and multivariate logistic regression model, that assess the difference in tick associated risk of multiple variables

Variables		Univariate Regression				Multivariate Regression			
				95% CI				95% CI	
		Sig.	OR	LB	UB	Sig.	OR	LB	UB
**Region**									
Reference	**Urban Areas**								
	**Environs**	<0.001	2.192	1.736	2.768	<0.001	1.979	1.538	2.547
	**Rural Areas**	<0.001	2.195	1.710	2.818	<0.001	1.965	1.492	2.589
**Age**									
Reference	**18–34 years**								
	**35–44 years**	0.254	0.854	0.651	1.120	–	–	–	–
	**45–54 years**	0.632	1.057	0.843	1.326	–	–	–	–
	**55–64 years**	0.833	0.977	0.784	1.216	–	–	–	–
	**≥65 years**	0.645	1.059	0.830	1.350	–	–	–	–
**Gender**									
Reference	**Female**	0.907	0.991	0.857	1.147	–	–	–	–
	**Male**								
**Profession**									
Reference	**Farmer**	<0.001	1.371	1.173	1.604	0.037	1.219	1.012	1.468
	**Forestry worker**	0.016	2.729	1.205	6.179	0.030	2.495	1.095	5.682
	**Horticulturist**	0.119	0.770	0.554	1.070	0.497	0.887	0.626	1.255
	**Other Outdoor worker**	0.940	1.019	0.628	1.652	0.737	0.919	0.559	1.510
	**Indoor worker**								
**Time spent outside**									
**Summer**									
Reference	**<1 h**								
	**1-3 h**	<0.001	1.959	1.458	2.632	<0.001	1.929	1.429	2.603
	**>3-6 h**	<0.001	1.889	1.420	2.512	0.002	1.595	1.184	2.147
	**>6 h**	<0.001	1.896	1.428	2.517	0.006	1.552	1.137	2.119
**Winter**									
	**<1 h**								
	**1-3 h**	0.343	1.108	0.896	1.370	–	–	–	–
	**>3-6 h**	0.155	1.187	0.938	1.502	–	–	–	–
	**>6 h**	0.253	1.166	0.896	1.519	–	–	–	–

*Sig* Significance, *OR* Odds ratio, *LB* Lower bound, *UB* Upper bound, *CI* Confidence interval

### Regional differences

**Fig. 1:**
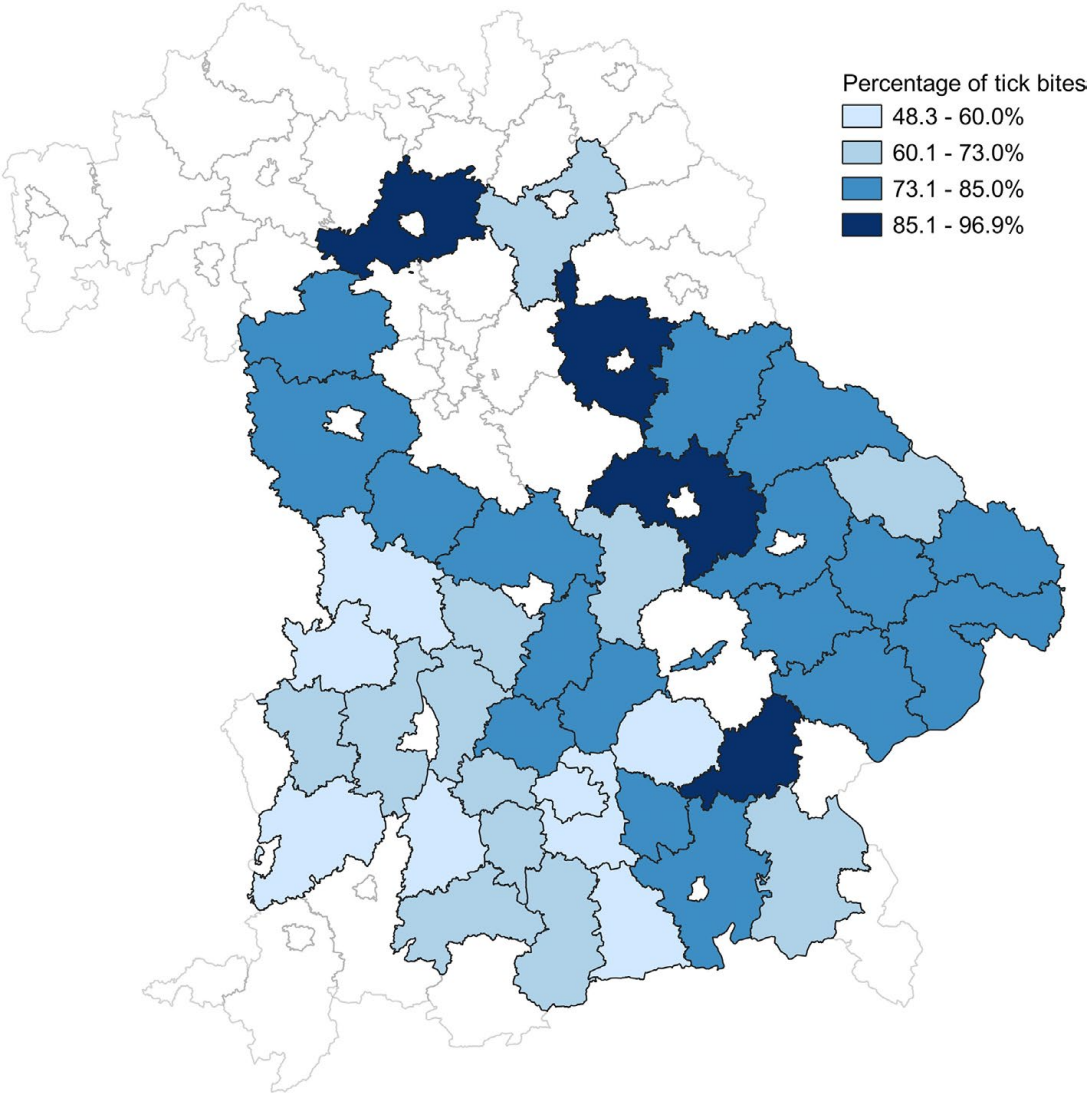
Percentage of people with previous tick bites in Bavaria in areas with 30 or more participants. The areas outlined in light grey represent Bavarian areas that were not included in the geographical analyses due to a low number of participants

Considering the areas in which more than 30 participants lived (*n* = 39) showed that the proportion of participants reporting at least one tick bite ranged from 48.3% in the area Landsberg am Lech (Bootstrap 95% CI: 37.0–60.0%) to 96.9% in the area Amberg-Sulzbach (Bootstrap 95% CI: 91.0–100%). In Munich, the only urban area considered in this analysis, 59.7% of people were affected. In the environs, the proportion ranged from 48.3 to 87.3%, with a mean of 70.2%. In rural areas, between 61.0 and 96.9% of people were affected, with a mean of 74.9%. Figure 1 shows that the proportion of affected people was slightly higher in eastern Bavaria apart from the areas of Ansbach and Neustadt a.d. Aisch - Bad Windsheim.

## Discussion

The aim of the study was to assess the prevalence of tick bites in various professions and regions in Bavaria. With a risk twice as high as that of indoor workers, forestry workers had the highest risk to have a previous tick bite followed by farmers. Furthermore, the highest proportion of affected people was found for residents of rural areas.

The study showed that certain outdoor workers had a higher risk compared to indoor workers. In line with these findings, prior studies also indicated a high prevalence of tick bites among forestry workers. It was found that forestry workers had a high seroprevalence of LB and other tick-borne diseases (TBD) as well as a background of high tick exposure at work and a self-reported history of TBD [33–36]. Since ticks are not only common in forests, the slightly lower risk for farmers could be attributed to modern agricultural methods with tall machinery that lead to less encounters with ticks attached to crops. Additionally, insecticides usage in agriculture might prevent farmers from receiving tick bites [37]. The lower risk of horticulturist could be explained by specific landscape management in gardens and thus, horticulturists ‘workspace, since the presence of ticks is limited by specific habitat requirements. Reduced tree canopy and residential lawns create poor environments for ticks as well as measurements such as leaf litter removal and > 1 m boarder between lawn and tick infested areas, and therefor decreasing the tick associated risk [7, 38].

The study results indicated that people from environs and rural areas are at an increased risk for tick bites. Proximity to nature in environs and rural areas may explain this increased risk. Several studies showed that tick-associated risk of LB or TBE was associated with close proximity to forests, a habitat abundant in *I. ricinus* [39–44]. Nevertheless, these findings are limited by missing information about leisure activities, personal protective measurements, and details about living conditions. In this study, we found that the tick-associated risk was influenced by the time people spend outdoors.

It was found that there was a slight increase in the proportion of affected people from south-western to eastern Bavaria. A similar pattern in TBE or LB cases was reported by the Robert Koch Institute and Böhmer et al. [6, 18]. This may be due to higher percentages of woodland areas, like the Bavarian Forest, in eastern parts of Bavaria [29, 45]. In addition, the highest number of previous tick bites from participant was found in Amberg-Sulzbach (96.6%), which was reported to be one of Bavarians TBE hotspots [4].

Since the analyses examined the tick bite prevalence only in areas with at least 30 inhabitants, findings are limited by the lack of data collected from northern Bavaria, where forested areas occupy larger stretches of land compared to in southern areas (pre-alpine areas not considered, as ticks are rare at altitudes greater than 450 m) [46, 47]. However, as 92 of the 96 Bavarian areas were declared risk areas for TBE, precautions such as education on prevention strategies and vaccinations are necessities in all parts of Bavaria. Prevention can include personal protection such as avoiding tick infested areas like high grasslands and areas of tree canopy, wearing protective clothes and repellants, and checking oneself for tick bites after returning inside and prompt removing of all found ticks. In parks and front yards, the risk can be decreased with lawn mowing and leaf litter removal [7]. Vaccinations are especially recommended for inhabitants of endemic areas, members of exposed occupation groups, and individuals in close contact with nature [18]. As vaccination rates have remained low over the last years, there may be lack of awareness of the importance of vaccinations for the prevention of the tick-borne disease TBE [48]. As ticks are dependent on the presence of appropriate hosts like deer and rodents, tick-associated risk can further be reduced by tick host management, through the use of controlled burnings and acaricides, which are also toxic for other potentially beneficial insects and mites [7]. While these methods were proven to be effective, they are less practical than vaccinations or personal protection strategies, of which the latter is critical for prevention of other tick-borne diseases like the bacterial LB.

There are some study limitations. As the largest study was performed at the ZLF, there was potential for a selection bias. Older, sick, or disabled people may have been less likely to attend the festival. Women were also slightly overrepresented, which similarly was the case for the study conducted in private practices in 2017. As self-administered questionnaires were used, desirability bias and recall bias are possible. Accordingly, the actual number of tick bites might have been over- or underestimated.

Although a large population was assessed, the generalizability is somewhat limited. For example, the mean age in this study is higher than the German average, as people had to be ≥18 years to participate [49]. Especially the proportion of middle-aged individuals aged 45–64 years was overrepresented compared to in the general population [50]. To evaluate the differences between residential areas, only areas with more than 30 participants were considered. Therefore, the low numbers of participants in some areas might have led to over- or underestimations. Bootstrap 95% CI was calculated to partly address this problem in areas included in our analysis.

## Conclusion

Despite these limitations, the large study population of 3503 participants demonstrates differences in risks for tick bites between various professions and residential areas. Prevention strategies need to be expanded, as reported numbers of TBE and LB cases are increasing in Germany, vaccination coverage remains on steadily low levels, and climate change supports environmental conditions in which *I. ricinus* thrives. Further studies focusing on personal protection strategies and awareness about the individual risk related to high-risk occupations and at-risk residential and geographic areas can be instrumental in strengthening primary and secondary prevention.

## Data Availability

The datasets used and/or analyzed during the current study are available from the corresponding author on reasonable request.

## Abbreviations

LB: Lyme borreliosis
TBE: Tick-borne encephalitis
OR: Odds ratio
CI: Confidence interval
ZLF: Bayerisches Zentral-Landwirtschaftsfest (Bavarian Central Agricultural Festival)
TBD: Tick-borne diseases
